# Primary health care providers’ views on managing substance use among people living with HIV

**DOI:** 10.4102/phcfm.v15i1.3984

**Published:** 2023-08-25

**Authors:** Ramprakash Kaswa, Marietjie de Villiers

**Affiliations:** 1 Department of Family Medicine and Rural Health, Faculty of Health Sciences, Walter Sisulu University, Mthatha, South Africa; 2 Division of Family Medicine and Primary Care, Faculty of Health Sciences, Stellenbosch University, Cape Town, South Africa

**Keywords:** primary health care workers, HIV, CHC, PLWH, adherence, substance use

## Abstract

**Background:**

The growing culture of substance use among people living with human immunodeficiency virus (PLWH) is a serious threat to the human immunodeficiency virus (HIV) pandemic. As the gatekeepers of comprehensive care, primary care providers are responsible for screening, assessing, and managing individuals who use substances.

**Aim:**

This study aimed to evaluate primary care providers’ views and approaches to substance use management among PLWH who attend primary care services in Mthatha.

**Setting:**

This study was conducted at Ngangelizwe and Mbekweni Community Health Centres (CHCs) in the Eastern Cape province’s King Sabata Dalindyebo (KSD) sub-district municipality.

**Methods:**

This qualitative phenomenological study involved the views of primary care providers. This study included 32 primary health care (PHC) providers. All participants were female except one male with a mean age of 48.6 years (range 27–64 years). Semi-structured interviews were conducted until saturation of the theme was reached. Then, the data from the transcribed interview were analysed with a thematic framework.

**Results:**

Substance use among PLWH was reported to be associated with poor clinical outcomes and disruption of antiretroviral therapy (ART) adherence. The significant barriers reported for substance use management in PHC settings were a lack of resources, skilled providers and poor community participation.

**Conclusion:**

Substance use management programmes are not commonly offered in PHC because of the lack of human and infrastructural resources, the lack of skilled providers and poor community engagement.

**Contribution:**

This study provides a context-specific PHC providers’ approach to substance use management.

## Introduction

The clinical outcomes of people living with human immunodeficiency virus (PLWH) have improved since the introduction of antiretroviral therapy (ART).^1^ It can also reduce the risk of human immunodeficiency virus (HIV) transmission if one sustains adherence to ART.^2,3^ In 2016, South Africa became the first country to make everyone with HIV eligible for treatment.^4^ Unfortunately, many still fail to take medicine or maintain optimal adherence.^5,6^ As a result, PLWH are experiencing high-risk behaviours and poor quality of life because of substance use.^6^ This issue is also linked to the development of suboptimum adherence to ART and reduced access to care. The most studied reported that poor ART adherence is caused by intoxication and forgetfulness after substance use.^5,7,8^

Primary care in substance use management refers to providing comprehensive and accessible healthcare services by primary care providers responsible for addressing a large population’s needs.^9^ They also develop long-term relationships with their patients. As the gatekeepers of comprehensive care, primary care providers are now expected to provide substance use services, including screening, assessment and managing substance-dependent patients.^9,10^ Early identification of substance use behaviour through screening in primary health care (PHC) settings creates an opportunity for optimum management. Like other chronic diseases, early screening detection can serve as a preventive measure and guide further clinical intervention where warranted.^11^ Substance use screening is part of the patient’s medical history in primary care to make the appropriate diagnosis, management and preventive care.^9^

Substance use screening in primary care can also benefit patients.^12^ It can help them identify their substance use problems and inform their healthcare providers about them. According to studies, asking patients about their substance use can increase their likelihood of discussing these issues with their primary care providers.^11^ In most cases, the relationships between the patients and the primary care providers allow for brief substance use assessments without hostility. One of the most critical advantages of substance use screening in primary care is that it can be followed up at subsequent visits.^13^

Although there are varying opinions about the appropriate use of substance use screening in a standard history, studies suggest that asking about the use of substances in the context of other lifestyle and behavioural questions is less threatening to patients.^11^ For instance, studies have shown that it is more acceptable for patients to undergo alcohol-related disorder screening if it is part of a comprehensive evaluation that includes other factors such as diet, exercise and medication use.^9,11^ In addition, asking about substance use in the context of other behavioural and lifestyle questions can help the patient and the primary care provider feel more comfortable, decrease anxiety and help the patient feel better about themselves.^11^ Finally, routine clinical care is widely believed to detect substance use. Still, the stigma surrounding this subject can make it challenging to measure substance use in standard clinical practice.^14^

Substance use has a more significant combined impact on the health and well-being of PLWH than any other preventable factor.^2^ Unrecognised substance use among PLWH significantly affects clinical, social and economic outcomes.^15^ As PHC is a gateway for community healthcare needs, many international public health institutes have supported primary care providers’ routine screening of substance use.^9,16^ A universal substance use screening is especially relevant in PHC settings where substance use is prevalent.^16,17^ However, only a few primary care facilities routinely screen for substance use among PLWH. Although it is widely believed that substance use can be measured in routine clinical care, it can be challenging because of the stigma associated with the topic.^14,16^

The use of substances has a significant impact on the engagement of individuals with HIV in primary care. This issue is a barrier to developing and maintaining a comprehensive HIV care system.^14,18^ Most of the evidence from quantitative studies describes the relationship between substance use and HIV care. Qualitative research can explore complex aspects of healthcare services among PLWH who use substances. It can provide more profound insight into primary care providers’ views on substance use management among PLWH. Primary health care settings offer an opportunity for a context-specific management approach for substance use among PLWH.^15^ Primary care providers can identify and intervene with PLWH who otherwise have no access to substance use management at specialist centres.^12,19^

This study is part of a larger project to evaluate the comorbidity of HIV and substance use and the response of primary care services to such patients in the Mthatha region of the Eastern Cape, South Africa. Few studies have explored the PHC provider’s role in substance use management in PLWH, with none from this particular context. This article reports the views and approaches of PHC workers on the current practices of substance use management among PLWH who attend PHC services in Mthatha.

## Research methods and design

### Study design and settings

Phenomenological interpretative methods were used to conduct this qualitative investigation. Through this method, the researcher focuses more on interpreting and comprehending a phenomenon or experience as lived. In this study, the researchers used this method to understand the individuals’ experiences better.

This study was conducted between 01 August 2018 and 31 October 2018, at King Sabata Dalindyobo (KSD) municipality in Eastern Cape province. The KSD is a rural area with about half a million population. Most of its residents are isiXhosa speaking. It is regarded as one of the most impoverished sub-districts in the country because of the high level of social welfare grants issued and the number of people using state facilities for healthcare.

Regional and central hospitals provide healthcare services in the KSD sub-district. There are also five community health centres (CHCs) and 42 clinics. Three CHCs are in the Mthatha township, while the others are outside. The biggest CHC is Ngangelizwe, which serves the Mthatha community. The smaller Mbekweni is 20 km away from the township.

The study population consisted of all healthcare workers involved in HIV care at CHCs in the KSD sub-district municipality. Human immunodeficiency virus care is not integrated into ambulatory primary care and is delivered through separate HIV clinics in all the CHCs. For example, at Ngangelizwe Community Health Centre, there were 52 healthcare workers, and 36 were involved in HIV care, including counselling, testing, initiation of treatment, and monitoring the linkage to care. At Mbekweni CHC, there were 38 healthcare workers, and 24 were involved in HIV care. There was one doctor in each community health centre. Other healthcare workers include lay counsellors, nurse assistants, enrolled nurse practitioners and professional nurses.

### Sampling and saturation of data

This study used stratified purposive sampling, in which participants met predefined criteria. The most prominent inclusion criterion was the participant’s experience with the phenomenon under investigation and those directly involved in the care of HIV patients. The five CHCs of KSD municipality were divided into two strata based on geographic location and the catchment areas of the population. One CHC from each stratum was selected for this study. In addition, we selected healthcare workers from two selected CHCs who provide HIV care to obtain a sufficient range of responses and experiences of the phenomenon under study. The sampling ended with confirmations of data saturation by carrying out two additional interviews with a repetition of findings from each CHC. Twenty interviews were conducted in Ngangelizwe CHC, and 12 interviews were conducted in Mbekweni CHC.

### Participants

In total, 32 interviews were held with healthcare workers from the two sampled CHCs. Twenty participants were from Ngangelizwe, and 12 participants were from Mbekweni. There were 31 female participants and one male participant with a mean age of 48.6 years (range 27–64 years). The cadres of health workers involved were 25 professional nurses, 5 enrolled nurse auxiliaries, 1 lay counsellor and 1 medical doctor. The doctor from Mbekweni CHC declined to participate in the study. The professional nurse provides various healthcare services, from chronic medical care to obstetric services at a CHC. They are responsible for developing and implementing a quality improvement plan. Besides providing effective and efficient care, the professional nurse delegates the nursing care responsibility to other healthcare providers. An auxiliary nurse is only authorised to provide primary nursing care when a patient’s care is based on a standard care plan. A professional nurse must supervise this type of care. The mean work experience of healthcare workers was 8.5 years (range 1–17). Table 1 demonstrates the demographic characteristics of the participants.

**TABLE 1:** Demographic characteristics of study participants (*n* = 32).

Characteristics of participants	Numbers
**Mean**	
Mean age	48.6 years
Mean work experience	8.5 years
**Gender**	
Male	1
Female	31
**Community Health Centre**	
Ngangelizwe CHC	20
Mbekweni CHC	12
**Profession**	
Professional nurse	25
Enrolled nurse auxiliary	5
Lay counsellor	1
Doctor	1
**Work experience (years)**	
Less than 5	9
5–10	13
More than 10	10

CHC, community health centre.

### Data collection instrument

Semi-structured interviews were conducted to gather qualitative data. A trained research assistant who has experience in qualitative research conducted the interviews. The research assistant was a registered student in psychology and from the IsiXhosa context. The purpose of the study was explained to the participants to maximise the response rate and generate reliable information. After obtaining written consent, a semi-structured interview was conducted. Most of the participants were from the IsiXhosa context. The participants were asked to choose their preferred language for the interview. The research assistant was able to speak both IsiXhosa and English fluently. Therefore, all the interviews were conducted in English as the medium the participants chose. Each interview lasted 30–60 min and was conducted privately in a secure consulting room. All efforts were made to conduct the interviews privately and without interruption to ensure that participants spoke freely. All interviews were audio-recorded and transcribed. The semi-structured interview guide is demonstrated in Table 2.

**TABLE 2:** Semi-structured interview guide overview of this study.

Theme	Primary question asked
Healthcare workers’ experience regarding substance use among PLWH	Describe your work with PLWH. Do you see PLWH who use substances? How was your experience?
Approach to substance use management among PLWH	What is your general approach to PLWH and who also uses substances? How do you find managing PLWH and who also uses substances?
Services for substance use management	What services is your facility providing to PLWH and who also uses substances?
Suggestions for improving substance use management	How can the management of PLWH and those who also use substances be improved? What would you like to see in an integrated management strategy for HIV and substance use at the PHC level?

PLWH, people living with HIV; PHC, primary health care; HIV, human immunodeficiency virus.

### Data analysis

Data were analysed with a thematic framework adopted from Braun and Clarke’s study on using thematic analysis in psychology.^20^ Firstly, the data were transcribed accurately from an audio-recorded interview. Secondly, transcripts were reviewed against the audio recordings for accuracy and completeness. Thirdly, non-verbal actions such as the lengths of pausing, coughing and tone of voice were described in detail using a formal transcription system. Fourthly, transcripts of the interviews were sent to the participants to enable them to correct their interpretation. Finally, all edited transcripts were uploaded into ATLAS.ti,^7^ a computerised qualitative data analysis software program for data management and coding. No names were recorded to ensure anonymity. Instead, the study participants were given the code number such as ‘P’ for participants, ‘N’ for Ngangelizwe CHC, ‘M’ for Mbekweni CHC, ‘1’ for a professional nurse, ‘2’ for an Enrolled Nurse Auxiliary, ‘3’ for a lay counsellor and ‘4’ for a doctor.

#### Step 1: Familiarising with data

The researchers reviewed the collected data independently during the study. The researchers thoroughly familiarised themselves with the data by reading and re-reading it actively to search for meaning and patterns. All the initial interests and thoughts were recorded with written notes. Analyses were conducted simultaneously with data collection, and inter-researchers’ differences were discussed. Data were interpreted in an iterative process according to established and accepted procedures for qualitative research.

#### Step 2: Generate initial codes

**FIGURE 1:**
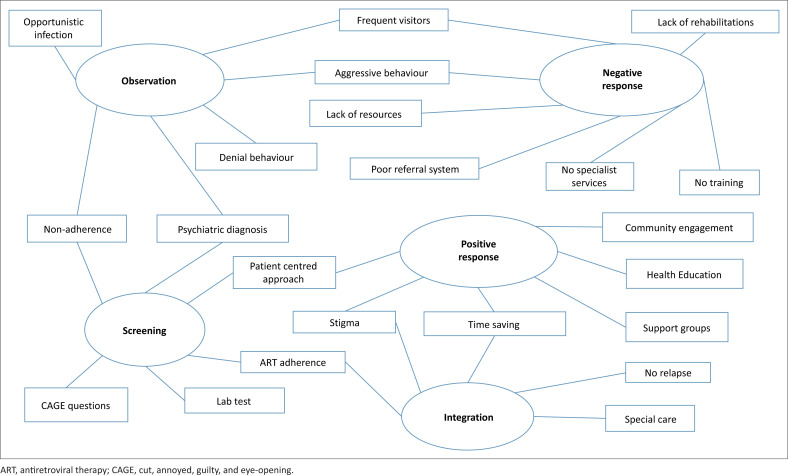
Initial thematic map of data analysis.

The repeated reading of the transcripts was used to develop a code list in the ATLAS.ti.^7^ Firstly, the emerging preliminary codes relevant to the study objectives were identified. Secondly, deductive codes from the data were added, and code lists were grouped, regrouped and refined, including primary and secondary codes (Figure 1). We then coded the various features of the data to generate initial codes. The coding process was conducted comprehensively, and each item was given equal attention.

#### Step 3: Search for themes

The themes generated by the process were inclusive and thorough. The researchers gather all the relevant data points for each potential theme independently. The themes concerning the coded extract were then checked. All the relevant extracts for each theme have been gathered. Themes were matched against each other and returned to the original data set. The themes were analysed and interpreted coherently. The data were then analysed and interpreted in a way that made sense. This step helps researchers develop a study’s thematic map, as demonstrated in Figure 1.

#### Step 4: Revisit themes

The data and the analysis themes were matched against each other, and the extracts provided a well-organised and convincing story about the topic and the data. There was also a balance between the illustrative and analytic extracts. Sufficient time was allocated for the various analysis phases to complete the task.

#### Step 5: Defining themes

The researchers then analysed the data to develop a clear and consistent set of terms for each theme. The last opportunity for analysis was the final analysis, which involved selecting the most compelling and engaging extracts from the study. This step helped the researchers create the final themes connected with the literature and research questions.

### Trustworthiness

To enhance the analytical rigour of the study, the researchers follow the Lincoln and Guba criteria.^21^ As a result, the researchers could form a coherent and effective data analysis by sharing the coding process and data analysis with the facility manager (nurse educator) and a clinical psychologist for independent inputs and discrepancies to be aligned in the coding process. In addition, the researchers determined the transferability by carefully analysing the various categories and subcategories of the study. We also discussed the research findings with four primary care providers who did not participate in the study and agreed with our results.

The researchers maintained a steady and reliable record of the interviews conducted during the study by recording and documenting them. Researchers also ensured that the entire process was monitored throughout the study period. Additionally, we ensured that the participants were informed about the various steps involved in the study. Finally, member checks were conducted with some participants to ensure the interpretation was valid.

### Ethical considerations

The research participants signed written informed consent after informing them about the project in a culturally and linguistically sensitive manner. The researchers took the necessary steps to ensure that the information was accurate and that the participants’ values and beliefs were not misunderstood. Furthermore, the confidentiality and anonymity of the participants were maintained. The Health Research Ethics Committee (HREC) of Stellenbosch University approved the study (HREC reference number: S18/01/001). Permission was also obtained from the Department of Health, Eastern Cape (National Health Research Database [NHRD] reference number EC_201803_007) and the local health authorities.

## Results

The qualitative data were categorised into key themes based on healthcare workers’ views, experience and issues regarding substance use management. The data collected from this study revealed three thematic categories of the experiences of healthcare workers involved in substance use management in PHC settings. The first theme focuses on the healthcare worker’s experiences with substance users. The second theme focuses on the management of substance use disorders. Finally, the third theme, which revealed the barriers to substance use management in PHC settings, consists of concerns from the study participants. Table 3 demonstrates the themes and sub-themes that emerged from the data on current practices of substance use management in primary care.

**TABLE 3:** Theme distributions of the current practices of substance use management at primary care.

Theme	Sub-theme
1. Experience with a substance user	Non-adherence to ART Withdrawal symptoms Poor outcome of comorbidities Deny behaviour and change providers frequently Psychiatric comorbidities
2. Approach to substance use management	Substance use screening CAGE questions Patient-centred approach Collateral and medical history Observation of behaviour Laboratory test Services offer for substance users Counselling Health education Community visits Support group Refer to a social worker and specialist
3. Barriers to substance use management	Integration of substance use management Community engagement Lack of training for healthcare providers Rehabilitation services Lack of resources (human and physical infrastructure)

ART, antiretroviral therapy; CAGE, cut, annoyed, guilty, and eye-opening.

### Experience with a substance user

#### Non-adherence to antiretroviral therapy

Most study participants cited substance use as the leading social deterrent for ART adherence among PLWH. In addition, participants have acknowledged that people who use substances always have issues with their treatment compliance. This viewpoint is captured in the following provider’s statement:

‘I think one of the other things is we always noticed that these people never finish their treatment … they always excuse for forgetfulness for treatment.’ (P4, Ngangelizwe CHC, Professional nurse)

Participants also highlighted the healthcare users’ concern about simultaneously mixing their medication with substance use. For example, one of the participants stated their viewpoint as follows:

‘They will tell you that last week he not ate any treatment because … [*H*]e spent their weekend with friends … you know, they are high on their stuff and cannot mix treatment with other stuff.’ (P5, Mbekweni CHC, Professional nurse)

In contrast, some other participants were not ready to endorse the effect of substance use if adherence to ART was maintained, and no adverse health issues were detected among healthcare users.

#### Withdrawal symptoms

Some study participants reported that their clients presented with withdrawal symptoms of substance use for medical attention. Unfortunately, binge drinking and drug overdoses are common among PLWH who did not disclose their status. Often, they use a substance as a coping mechanism and are more frequently presented with emergency medical attention for their substance withdrawal symptoms. This concern was expressed by a participant who noted that they were worried that a patient might have emergency medical conditions or immune reconstruction inflammatory syndrome (IRIS). They subsequently learned that the client was registered at the antiretroviral (ARV) clinic but never disclosed substance use, resulting in a drug dependency withdrawal. Her view is as follows:

‘Think a person in particular who comes in, and by the time you talked about his pain … he demands an injection for pain … one can see their clinic cards … several times he came for the same injection … and target new healthcare providers.’ (P9, Ngangelizwe CHC, Doctor)

#### Poor outcome of comorbidities

Most participants believed substance use is a social determinant of health that can affect a person’s response to treatment and risks of disease progression. According to them, people who use illicit substances are more likely and frequently visit an emergency clinic than the general population. They also highlighted how a lack of knowledge about this issue could affect their decisions regarding treatment. Substance use affected ART compliance and brought a range of risky behaviours, such as unsafe sexual practices and social and legal issues. Most agree that people who use substances visit health facilities for recurrent medical attention. A participant shared this view:

‘These people never do well despite all our effort … then you found that they mess with drugs. so if I saw a person not doing well on ART, I always look for substance use.’ (P8, Ngangelizwe CHC, Professional nurse)

#### Deny behaviour and change provider frequently

The participants noted that some individuals might not be ready to share their substance use problems. Despite knowing they are using a substance, they do not feel comfortable disclosing it. Often, people do not commit to receiving help for their substance use problems from their providers; this issue was reported as more common among males. Some said their substance use responded to their frustration and confusion related to HIV. In addition, substance use is viewed differently in terms of stigmatisation. Some people feel they are unfairly blamed for their substance use disorder; others are denied. The following statement from one of the participants exemplifies this theme:

‘If one is still addicted and denies their use of alcoholism or other drugs or whatever it is … you know, they move from clinic to clinic … you can see their last six-month treatments from more than two different clinics.’ (P4, Mbekweni CHC, Enrolled nurse auxiliary)

#### Psychiatric comorbidities

Most participants said that patients who use substances have one or more psychiatric conditions during their lifetime. According to them, the comorbid psychiatric diagnosis makes things more complicated regarding their substance use management. One often gets poor cooperation from these patients regarding compliance with treatment for both conditions. The following quotation from providers supports this theme:

‘These people have a lot of psychiatric problems … However, those are just difficult for us …. you know, we spent lots of time shortening their issues. So you need like a full dedicated team when that comes up.’ (P9, Mbekweni CHC, professional nurse)

‘They come with all these abnormal and aggressive behaviors complain but the end of the day, and you find that it all starts after drugs.’ (P9, Ngangelizwe CHC, Doctor)

In addition, participants also reported that dealing with patients with psychiatric disorders was challenging. Substance use and HIV both adversely affect the outcomes of psychiatric diseases. Therefore, they always need a multidisciplinary specialist team for management; a resulting willingness to work with these individuals varied among participants. Most participants expressed that they need a higher level of care than CHCs.

### Approach to substance use management

#### Substance use screening

Most participants reported that they would often ask about a patient’s substance use during their initial visit, especially when they sought help for a specific health issue. However, according to them, this approach was unrealistic as it could lead to inaccurate information. For instance, it could be challenging for patients to disclose their substance use because their use patterns change over time. Most support using substance use screening tools to identify patients at risk of developing substance use disorder. They noted that it could help them understand their condition and intervene accordingly. Some also believe it can help them treat their patients more effectively:

‘Sometimes, they do not even realize that they are in trouble. So maybe just talking about … how much they drink or smoke unless someone calculates for them … you know, the CAGE question helps in this regard.’ (P3, Mbekweni CHC, Enrolled nurse auxiliary)

Although there was a disagreement about how to conduct screening, most participants preferred a self-administered tool. Most participants agreed that substance use screening should be essential to a patient’s medical history. According to most participants, this method could save them time and effort. Another reason was that people were more likely to disclose their substance use more comfortably:

‘I think people often do not know where to start and what to talk about. So I think it is important for us to give people that opportunity … It’s also to understand better whom you are working with … so we prefer to hear from their mouth.’ (P3, Ngangelizwe CHC, Enrolled nurse auxiliary)

Some of them agreed that it should be done with more comprehensive approaches. During the screening process, sensitivity must be emphasised over the specificity of the results. It is more important not to miss an actual case than it is to identify patients who do not have a substance use disorder. A participant summarised this perspective:

‘Sometimes these tools do not help at all … especially when they are in denial … you have to dig deep … you know to reach out to family and friends.’ (P2, Mbekweni CHC, Professional nurse)

People with substance use disorders often do not feel comfortable being judged. They do not want to be put through the process of being evaluated. Some participants said they often run urine tests to convince patients that drugs are in their bodies. Some participants even preferred this method because it could save time:

‘With the substance use, people are not comfortable being judged … you rarely get them in a first visit … you know, I guess societal expectations or whatever … you need to observe their behavior consistently … sometime we run few tests on their urine to convince them.’ (P12, Ngangelizwe CHC, Professional nurse)

#### Services offered for substance users

Various factors can affect the development and maintenance of substance use disorders. Besides genetic, medical and psychological factors, other environmental elements such as family, social and cultural factors can also contribute to the development of the condition. Therefore, a team effort is needed. The advances in behavioural therapy over the last few decades have led to advancements in treating various illnesses. These include health education, counselling and behavioural change therapy, which can be used to treat substance use disorders. Most participants reported providing basic health education and counselling for all patients. However, only a few reported brief behavioural change counselling for substance use management. One of the participants stated their viewpoint as follows:

‘Adherence to ART among HIV patients is a big challenge for these people … we offer health education and adherence counseling for them every visit.’ (P7, Ngangelizwe CHC, Enrolled nurse auxiliary)

Lack of time is a significant factor that prevents them from addressing substance use in their primary care settings. In addition, they noted that there are usually multiple priorities when addressing the issue:

‘The substance abuse issue in these people is not as straightforward as it looks … there are a lot of psychosocial issues that are behind it … if one wants to deal with it, you need to work with patience … and yes, of course, offer counseling.’ (P7, Mbekweni CHC, Professional nurse)

According to participants, primary care visits can help patients develop a plan to prevent substance abuse. They also believe this process can help them identify their potential risk-taking behaviour. During a primary care visit, providers can also educate their patients about the importance of maintaining a healthy lifestyle and preventing substance abuse:

‘First thing every morning, we talk about lifestyle, health education, and promotion in group interaction with our clients … the drug abuse is part of it … but we are unsure how far our massage goes.’ (P1, Mbekweni CHC, Professional)

Most participants echoed that service providers must consider an appropriate referral when addressing comprehensive substance use management needs. Psychological therapies could play an essential role in the recovery process because they focus on the various factors contributing to an individual’s substance abuse. These therapies helped identify the root cause of the issue and prevent the person from developing a negative mindset. This approach could help them provide their patients with the necessary support and resources to meet their needs:

‘People who develop drug dependency usually refer them to our psychologist and sometimes to the hospital for specialist care.’ (P1, Ngangelizwe CHC, Professional nurse)

Few participants mention community outreach for health education and catch-up plan for different programmes and substance use management. The following quotation expresses the view of one of the providers:

‘Once a while in a month, we visit our surrounding community as an outreach program. There are some support groups in the community … we often involved them.’ (P5, Ngangelizwe CHC, Professional nurse)

### Barriers to substance use management

Although there was broad agreement on aspects of the substance use management process, there were varying opinions on others. Therefore, the participants’ views on the barriers of substance use management in primary care are further categorised under the following subthemes.

#### Integrated management of substance use

Although most providers support integrating substance use management into their primary care, few raised concerns. According to most participants, the lack of time is one of the main barriers to addressing substance use in the community. In addition, they noted that there are multiple competing priorities in the care of these patients, and integration is the solution for managing all preferences in one consultation. Therefore, they felt that the lack of integration hinders substance use management among PLWH. On the contrary, some participants raised concerns that having this type of integration in a primary care setting is not ideal. They also believe that this process can be time-consuming and require special care. However, most providers noted that they would like to see more primary care-integrated services in the future. The views of one such participant were captured in the following statement:

‘They are a high-risk population with special needs … you cannot simply mix them with other patients.. other things are they come with many other health issues such as psychological and mental illness. Furthermore, those are very time-consuming … So you need to spend lots of time with these clients.’ (P15, Ngangelizwe CHC, Professional nurse)

**FIGURE 2:**
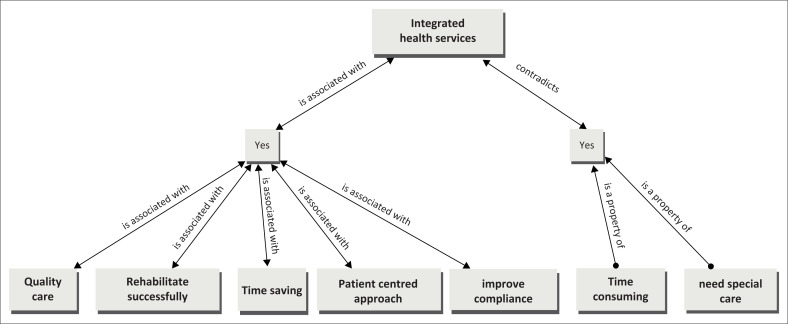
Primary health care providers’ views on integrated management of substance abuse.

Figure 2 captures the views of primary care providers for the integrated management of substance use.

#### Community engagement

Despite the positive effects of engaging with community resources, many barriers still prevent people from recovering from substance misuse. Most participants highlighted the importance of a community-based treatment approach for substance use. It focuses on providing a continuum of care to individuals struggling with substance use disorder. It involves coordinating multidisciplinary services to meet the needs of the patients. This management approach provides services that include screening, diagnosis, treatment and outreach. This view is summarised by one of the participants:

‘I think our community is struggling with this drug problem … you see these young kids from school experimenting different kinds of stuff and ending here … if you want to win this war, then you have to engage with community … otherwise you treat one tomorrow five will come.’ (P2, Mbekweni CHC, Professional nurse)

#### Lack of training for healthcare workers

According to most participants, the lack of resources for treating substance use disorders is a significant issue affecting these patients’ quality of care. They stated that better systems must be implemented before implementing integrated substance use management in primary care. In addition, participants noted that they do not have the necessary skills to identify and appropriately manage substance use disorders. However, a few of the participants acknowledged the training received in substance use management. They still need additional hands-on training to be more confident in addressing the issue. The viewpoint of one of the participants is as follows:

‘Sometimes you are not justifying them … you know, when you do not have proper training … you find yourself in this place… Hopefully, we will have some training and counseling resources … but most of our clinics do not have them.’ (P10, Ngangelizwe CHC, Enrolled nurse auxiliary)

Healthcare providers highlighted might not have the necessary skills and training to help their clients with substance use disorders. Some even said they do not regularly screen their patients because they do not know how to manage a positive screening for substance use. Some participants argued that substance use treatment needs a specialised referral centre, and none is available close to our CHCs. This viewpoint was shared by one of the providers:

‘We can talk about substance abuse issues, but to have someone to do the kind of follow-up … we love to have a place where one can specifically refer the severe cases … so that they can get them to a place where they can attend by a specialist in that field … otherwise they come again and again.’ (P8, Ngangelizwe CHC, Professional nurse)

Some participants, however, hope that the increasing number of substance users in primary care might shift the training of substance use disorder from specialised centres to primary care.

#### Rehabilitation services

Primary care providers can play a vital role in addressing substance use. Both primary and secondary prevention can be initiated in primary care. On the contrary, tertiary prevention is when a patient needs specialised services such as a rehabilitation centre. Most participants from both health centres mention that there are no rehabilitation centres in the region where they can refer to severe cases of substance dependency. As stated by one of the providers:

‘[*F*]or smoking, we have pretty options … like health education and counseling them about it … However, the problem comes when someone on alcohol, drugs, and other stuff … have no idea where to send them … there is no rehab in this area.’ (P9, Ngangelizwe CHC, Doctor)

#### Lack of resources (human and infrastructure)

According to most participants, resource-equipped systems are needed to manage patients with substance use disorder. Participants from both CHCs reported that lacking human resources is the most significant barrier to adequately working with such patients. They also highlighted the infrastructure constraints of overcrowded health facilities. They stated that it is challenging to maintain patient information privacy when you share a consulting room with other care providers and patients. As a result, they cannot connect patients with the appropriate services. One participant stated that it is discouraging to feel unable to provide adequate care. The following quotation highlights this theme:

‘And we feel like the limited with both human resources and space for handling a such high number of patient who is looking for these services … we hardly have one or two people on the ground to look after of these huge number of people come for chronic medication … and then you get them on top of that … you know we are trying, but resources are not on our side.’ (P5, Ngangelizwe CHC, Professional nurse)

## Discussion

This study investigated PHC workers’ views on substance use management in primary care settings. The results assisted in gaining a deeper understanding of the various factors that affect the quality of care for people with substance use disorders, specifically in an impoverished rural population. For example, most participants felt active substance use is linked to a lower possibility of starting and sustaining ART. This is similar to findings reported from the literature that substance users often fail to maintain a high level of ART adherence, which could worsen their medical conditions.^17,22,23^ Another factor influencing the course of treatment is the person’s beliefs about the interaction between substances and antiretroviral drugs. These beliefs can prevent people from fully participating in the HIV care system and accessing their medications.^2,18,24^ This could result in suboptimum adherence or completely undermine the effectiveness of their treatment.

Some providers noted that clients use substances to manage their anger and confusion. Social stigma prevents people with substance use disorder from seeking help. It makes them feel like people are judging them.^11,25^ Several studies also found that substance use behaviours were associated with psychosocial distress and mental health problems among PLWH.^26,27^ In addition, individuals with HIV-related issues described various coping adaptions, such as denial and avoidance.^25^ The findings from this study are consistent with the literature that substance use is a highly stigmatised health condition.^14^ As a result, our findings support community awareness and health education about the importance of substance use management to address its effects on HIV treatment.

Although there are varying opinions about the effectiveness of screening for substance abuse, this study concluded that it is not harmful to patients to ask about their substance use. There is an opportunity to address this issue in primary care because substance use is linked to higher-risk behaviour and poorer quality of life.^26,28,29^ Therefore, screening substance use in primary care is vital for comprehensive management. Substance use screening aims to identify individuals who may be at risk and address such harmful behaviour.^11,14^ Findings from this study also support that substance use screening is an important determinant of the safety and quality of healthcare. It is also essential to a patient’s medical history and can help make accurate diagnoses and management.

Substance use screening can help to understand a patient’s overall health and provide necessary information to make informed management decisions.^9^ Some study participants also noted that it could be very beneficial to understand the patient’s social determinants of health.^26^ Our study reported a lack of uniformity in primary care for substance use screening. It was also evidenced by some providers’ lack of reporting substance use.^28^ Some participants have raised concerns about the potential adverse effects of screening on their patients. In our interviews, many participants said patients would not disclose information about their substance use unless they were assured they would not feel threatened or judged. The study findings support the need for universal screening for substance use among PLWH who attend primary care.

The actions taken after a positive substance use screening result depend on the issue’s severity and the available resources.^9^ Other factors, like the type of assessment and the primary care providers’ expertise, are also considered.^30^ In our study, primary care providers hardly use brief interventions to help patients improve their substance use behaviour. During these visits, the patient should be provided with information about the screening results, the safe consumption limits and the strategies for change. A brief intervention costs relatively low, as it involves only one to two visits and a limited number of sessions.^14^ Usually, a follow-up visit is recommended for patients with serious problems. The number of sessions and the frequency of visits depend on the patient’s response and the issue.^9^ A brief intervention aims to help patients reduce their alcohol or drug consumption and minimise their associated problems. The intervention can vary depending on the patient’s current condition and previous attempts at treatment. For some patients, it can be used as a primary prevention tool for those at risk of developing problems; for others, it could be part of their management intervention.^14,31^

Supporting people with diverse needs requires strong links to primary care and substance use management services.^13^ Primary health care settings can manage substance use by integrating its management with HIV care.^13,26^ Having integrated substance use services in primary care can improve access to treatment for people who may not be able to receive it because of their primary care status.^9^ It can also help to enhance the continuity of care and successful rehabilitation.

Although integrating substance use services into the PHC system could positively impact people’s quality of life, these services were largely absent in our study. The task shifting from treating speciality mental disorders to treating non-specialist health workers has been widely used to expand access to mental healthcare and substance use services.^11,14^ This strategy could be implemented in a primary care setting where mental health practitioners are limited. In South Africa, task shifting from treating specialists to treating primary health providers has been considered a strategy to expand access to substance use services in primary care settings.^9^ Unfortunately, the implementation of this strategy has been hindered by the lack of definitive answers regarding the appropriate integration of substance use services into the PHC system.

Substance use behaviour can also be managed successfully through the intervention of primary care providers.^32,33^ The main barriers to substance use treatment were lack of resources, inadequate space in a health facility and the time constraints of primary care visits.^29,32^ Care providers often raise concerns about their patients’ time and competing demands during primary care visits. They also raised concerns about the lack of necessary knowledge about the various aspects of substance use.^14,34^ For example, in our study, providers suggested that a positive screening could lead to a referral to a speciality care facility. Still, they did not mention the primary care provider’s role in managing substance use disorder. This issue could be related to the lack of awareness about the skills needed for substance use management.^9^ We believe that training on substance use management can improve their skills in conducting motivational interviewing, which can help individuals with substance use to elicit their behavioural change.^26^ Having the necessary knowledge and resources to identify and provide effective interventions could also improve the quality of care for those with HIV.^26,32^

Finally, the relationship between healthcare providers and their patients is significant in maintaining an effective healthcare system.^26,28,32^ Therefore, how the screening questions are asked is more important than the person asking. For primary care screening, the tool’s sensitivity should be emphasised over the specificity of the results. It is essential not to miss cases than those who do not have a substance use disorder.^14^ For instance, a study revealed that the cut, annoyed, guilty, and eye-opening (CAGE) questionnaire’s sensitivity was enhanced by the open-ended question, which made it easier for researchers to track the participants’ substance use.^11^

On the contrary, illegal substance users and problem drinkers may feel embarrassed by their responses and often respond with hostility.^11,18^ To avoid making patients feel embarrassed, providers should pose questions non-judgementally. They should also accept honest responses without judgement. Programmes must also be designed to meet the needs of individuals who use substances. It can be done by establishing an integrated substance use management strategy at the primary care level that focuses on addressing the needs of these marginalised groups.^14,28,35^ The study results provide a framework for implementing a comprehensive substance use management strategy in PHC settings. It also suggests that providers should have the necessary skills and resources to manage substance use disorders.

### Limitations of this study

While this was a small study based in one geographical context, it is one of a few conducted in a rural population in South Africa. While we included two CHCs, most participants were female except one male provider. The male provider could have different views of substance use management. In addition, the limited work experience of some participants could have influenced their contribution to the study results. Most were nurses, and other healthcare providers’ views might not be well represented. However, the providers that agreed to participate may represent a subgroup of highly motivated providers to address substance use, and individual participants’ pessimistic attitudes towards substance use cannot be excluded. This study did not perform cross-case and within-case data analysis that handled the case’s unique attributes and patterns. Although the research was conducted through independent interviews, the study also considered reflexivity and researcher bias. The lead researcher is a primary care provider who also provides healthcare services to a select group of CHCs.

## Conclusion

Substance use is the primary social deterrent to ART adherence. Despite the universal screening strategy for substance use, only a few primary care providers utilise these services regularly. The lack of community engagement, shortages of skilled providers and the lack of health infrastructure are significant barriers to substance use management services in primary care. Appropriate training and a patient-centred integrated primary care approach can help substance use management among PLWH.
